# Introducing Compassionate Care to Undergraduate Health Science Students and Evaluating Its Implementation: A Pilot Study

**DOI:** 10.7759/cureus.104092

**Published:** 2026-02-23

**Authors:** Spyridon Rigatos, Christos Lionis, Mary Gouva, Anastasios Tzenalis, Marilena Anastasaki, Eleni N Albani

**Affiliations:** 1 Department of Nursing, University of Patras, Patras, GRC; 2 Department of Social Medicine, Faculty of Medicine, University of Crete, Heraklion, GRC; 3 Scientific Laboratory of Psychology and Person-Centered Care, Department of Nursing, University of Ioannina, Ioannina, GRC; 4 Laboratory of Child Care and Family Resilience, Department of Nursing, University of Patras, Patras, GRC

**Keywords:** asynchronous learning, compassionate care, digital tools, empathy, healthcare education

## Abstract

Introduction: Healthcare education faces a documented "empathy crisis," where students often experience a decline in empathic capacity during clinical training. While compassionate care is fundamental to patient outcomes, it is frequently sidelined in traditional biomedical curricula. Asynchronous digital pedagogy offers a potentially scalable, exploratory solution to bridge this humanistic gap.

Aim: This pilot study aimed to evaluate the potential impacts of an asynchronous, distance-based educational intervention in enhancing empathy and compassionate care among undergraduate healthcare students from the Departments of Nursing, Physiotherapy, and Medicine.

Methodology: A pilot pre- and post-intervention study was conducted with 50 undergraduate healthcare students. In the absence of a control group, the study utilized a convenience sampling design. The intervention consisted of a five-module asynchronous digital course focusing on applications of compassionate care. Empathy and compassion competence were assessed using the Toronto Empathy Questionnaire (TEQ) and the Compassion Competence Scale (CCS). Statistical analysis was performed using non-parametric tests, including the Wilcoxon signed-rank and McNemar tests, along with Spearman's rho correlation and Bonferroni adjustments for subgroup comparisons.

Results: Statistically significant improvements were observed across all domains. Median total empathy scores (TEQ) increased from 47.00 to 55.00 (p<0.05), while the proportion of participants reflecting higher-than-normal empathy rose from 58% to 88% (p<0.05). Significant increases were also recorded in all compassionate competence dimensions: Communication, Sensitivity, and Insight. A notable "convergence effect" was observed, as Medical and Physiotherapy students, who initially presented lower baseline scores, exhibited pronounced improvements, suggesting that the intervention may help narrow inter-faculty competency gaps.

Conclusions: The findings suggest that structured, distance-based compassionate care training is associated with positive impacts on empathy and compassion-related competencies.

## Introduction

In the contemporary healthcare landscape, the delivery of high-quality patient care transcends technical proficiency, necessitating a deep integration of compassionate care and empathy as core clinical competencies [1]. While these terms are often used interchangeably, they represent distinct psychological and professional constructs. Empathy involves the cognitive and affective capacity to understand the patient’s internal perspective. Compassion extends this resonance into an intentional response to suffering, characterized by a desire to alleviate distress, and compassion competence represents the professional ability to effectively translate these internal states into measurable clinical behaviors [2]. Research consistently demonstrates that empathetic engagement is positively correlated with enhanced patient satisfaction, improved diagnostic processes, and clinical decision-making quality [3].

However, as healthcare systems move further into a digital technology-driven age, the humanistic element is often challenged. The delivery of compassionate care faces unique hurdles in an era of rapid digitalization, where clinical detachment is frequently observed [4]. Despite these challenges, compassion remains a fundamental pillar of therapeutic success, potentially associated with reduced stress-related physiological responses and improved health outcomes [5].

A critical challenge in healthcare education is the documented "empathy decline" among undergraduate students during clinical training [6]. While widely reported, recent literature suggests this phenomenon is heterogeneous, with its trajectory varying across different cultural contexts and measurement tools. This erosion is often attributed to a "hidden curriculum" where institutional pressures and the dominance of the biomedical model prioritize efficiency over human connection. Evidence suggests that targeted training can mitigate this effect, enhancing empathic performance as perceived by standardized patients even when self-reported measures fluctuate [7].

Traditional undergraduate programs often lack formal, structured courses on relational skills, leaving students ill-equipped to handle the emotional labor of clinical practice [8]. Furthermore, the absence of training in self-compassion and resilience contributes to rising rates of professional burnout. This gap necessitates a shift toward human-centered educational models, such as the Attitude, Ethics, and Communication (AETCOM) frameworks increasingly adopted in global medical curricula, which prioritize longitudinal humanistic training.

To address these deficiencies, the development of digital educational environments offers a transformative solution. Modern educational technology, grounded in constructivist learning theories, allows students to actively construct knowledge through interaction with digital content [9]. By utilizing Project-Based Learning (PBL) and digital storytelling, educators can foster critical thinking and empathy [10]. Narrative Medicine serves as a powerful model for developing reflection; by engaging with patient stories, students learn to represent and respond to the suffering of others [11]. Digital tools provide a "safe-to-fail" environment where students can practice complex interpersonal skills [12]. Asynchronous modules are particularly effective for modern learners, supporting the principles of repetitive practice and self-paced reflection [13].

Given the urgent necessity to bridge the gap between theoretical humanism and clinical practice, this pilot study aims to evaluate the implementation of a specialized asynchronous online platform designed to introduce compassionate care to undergraduate healthcare students in Greece. The curriculum was based on content previously validated through an e-Delphi consensus study [14]. We hypothesized that this asynchronous digital intervention would be associated with significant enhancements in students' empathy and compassion competence, providing a potentially scalable and standardized solution to address current curricular deficiencies in healthcare humanism.

## Materials and methods

Setting and period of intervention

The pilot study was conducted within the framework of a research project supported by the 'C. Caratheodori' internal grant program (grant no. 81840) and took place from October 2025 to January 2026.

Type of research

A pilot, pre- and post-intervention design without a control group was employed to evaluate the potential impacts of the educational intervention on compassionate care and empathy.

Sampling and participants

A convenience sampling method was used to recruit undergraduate healthcare students. Recruitment was conducted between October 2025 and January 2026 through informative announcements posted in public digital communities and official university groups for students of Nursing, Physiotherapy, and Medicine. A total of 50 undergraduate students participated in and successfully completed the entire educational cycle. The sample consisted of 25 (50%) Nursing students, 15 (30%) physiotherapy students, and 10 (20%) medical students. Given the pilot nature of the study, the sample size was determined by voluntary enrollment during the academic period, without an a priori power analysis.

Intervention

The intervention consisted of an original asynchronous online course titled "Compassionate Care," hosted on a dedicated learning management platform (https://compassion.upatras.gr/). The curriculum was based on validated content [14] and structured into five thematic modules: Introduction to Compassionate Care, Theoretical and Therapeutic Approaches of Empathy and Compassion, Practicing Compassionate Care in Clinical Settings, Self-care and Resilience, and Narrative Medicine and Creative Applications. To ensure high levels of participant engagement and reproducibility, the platform was accessible to participants from October 2025 to January 2026, allowing students to navigate the material at their own pace according to the principles of asynchronous learning. The modules comprised a total of 13 specialized chapters, utilizing animations, educational games, and case studies to facilitate an immersive learning environment. To address the inherent limitations of distance-based education and maintain a "human touch," students were integrated into professional communication groups moderated by the lead researcher for reflective dialogue. Mastery of the curriculum and monitoring of engagement were ensured through mandatory quizzes at the end of each of the 13 chapters, each consisting of 15 questions. Students were required to achieve a minimum passing score of 80% to unlock subsequent chapters, a mechanism designed to guarantee that all participants engaged thoroughly with the core conceptual material and attained a consistent level of comprehension before proceeding to the next phase of the pilot project.

Data collection techniques

The data collection followed a structured three-step process. In the initial assessment (pre-phase), before accessing the platform, participants completed a Google Form containing demographic questions and two validated psychometric scales. Compassion was evaluated using the Compassion Competence Scale (CCS), employing the Greek version validated by Klimentidou et al. [15], while empathy was measured using the Toronto Empathy Questionnaire (TEQ), applying the Greek translation and validation by Kourmousi et al. [16]. Written permission for the use of these Greek versions was obtained from the respective researchers. The original English versions of the instruments, from which the Greek scales were derived, are provided in the Appendix for reference, with proper attribution to the original authors. The TEQ is a self-report instrument that assesses empathy as a primarily emotional construct, providing a robust measure of the participants' empathetic capacity. The internal consistency of the scales was verified in the current sample, with Cronbach’s alpha values indicating good to excellent reliability (range: 0.75-0.91). During the implementation phase, participants navigated through five thematic modules, where a mandatory quiz followed each chapter to reinforce learning and monitor engagement. Finally, in the post-phase assessment, upon completion of all modules by January 31, 2026, students completed a follow-up Google Form to measure changes in the same scales that had been assessed at baseline.

Ethical considerations

The research protocol was formally submitted to the Research Ethics and Deontology Committee (E.H.D.E.) of the University of Patras and was assigned protocol number 12169. The committee ruled that the study did not require further formal review, as it involved non-invasive educational research within the framework of the "C. Caratheodori" research project (grant no. 81840). All participants were fully informed about the study’s purpose and the voluntary nature of their participation. Electronic informed consent was obtained from all students prior to their participation. To ensure confidentiality, all data were collected anonymously, and no personal identifying information was stored or linked to the responses.

Data analysis

In the current pilot survey, data were analyzed using IBM SPSS Statistics for Windows, Version 26 (Released 2019; IBM Corp., Armonk, New York) and Python programming. First, reliability statistics were performed using Cronbach’s alpha, while descriptive statistics, including frequencies, percentages, means ± standard deviations (SD), and medians (interquartile range, IQR), were computed to summarize participants’ demographic characteristics and research measurements. A detailed descriptive analysis of individual questionnaire items was also conducted. The normality of continuous variables was assessed using the Kolmogorov-Smirnov and Shapiro-Wilk tests; due to violations of normality (p<0.05), non-parametric statistical tests were employed.

For pre- and post-intervention comparisons of paired data, the Wilcoxon signed-rank test was used to examine changes in total empathy and compassion competence scores for the entire sample and per university faculty group. Effect sizes for these comparisons were calculated using the r statistic. Additionally, the McNemar test was applied to paired categorical data to assess changes in the proportion of participants above the empathy cut-off score. Associations between pre- and post-intervention measurements were explored using Spearman’s Rho (ρ) non-parametric correlation.

To account for the risk of Type I errors, the analysis was strictly partitioned by scale and subscale, treating each construct as a distinct primary outcome. Furthermore, multiple comparisons were performed using the Bonferroni correction method specifically for the subgroup analysis examining the effect of the university faculty group on each scale and subscale, respectively. Statistical significance was set at α=0.05 for all analyses, and effect sizes were interpreted according to conventional benchmarks to assess practical significance alongside statistical probability.

## Results

Demographics

A total of 50 undergraduate healthcare students completed the pre- and post-intervention assessments. The majority of participants were female (37, 74%), while 13 (26%) were male. The age range was 18-25 years, and all 50 (100%) participants were of Greek nationality and enrolled in a Greek public university. Regarding academic distribution, 25 (50%) were students of the Department of Nursing, followed by 15 (30%) from the Department of Physiotherapy and 10 (20%) from the Department of Medicine. The cohort consisted primarily of senior students, with 26 (52%) in their fourth year or higher and 21 (42%) in their 3rd year. Most participants (46, 92%) were not employed in the health sector at the time of the pilot study. Geographically, 40 (80%) resided in urban areas. Notably, 47 (94%) of the sample had no prior exposure to seminars or courses regarding compassionate care, highlighting the educational gap addressed by this intervention.

Comparison of pre- and post-intervention scores

Baseline assessments indicated that participants’ scores on the TEQ and the CCS were within the moderate range. Prior to the intervention, more than half of the students exhibited higher-than-normal empathy based on the established cut-off score. Similarly, compassion competence was moderate across the Communication, Sensitivity, and Insight subscales, suggesting a baseline proficiency in compassionate behaviors that left room for further educational development (Table 1).

**Table 1:** Scores of TEQ and CCS measurements per assessment phase (pre and post) among 50 healthcare students in Greece. Descriptive and inferential statistics for empathy and compassion competence. Sources: The Toronto Empathy Questionnaire (TEQ) is adapted from Spreng et al. (2009) [17], and the Compassion Competence Scale (CCS) is adapted from Lee & Seomun (2016) [18]. The validated Greek versions [15,16] were used with written permission obtained from the original authors. Original English items for both instruments are provided in the Appendix for reference.

Measurements	Pre-Phase					Post-Phase				
	Mean ± SD	Md (IQR)	Min	Max	p-value	Mean ± SD	Md (IQR)	Min	Max	p-value
TEQ	45.82 ± 9.46	47.00 (36.00–54.00)	29.00	61.00	<0.05	53.22 ± 7.09	55.00 (50.00–58.00)	31.00	62.00	<0.05
CCS-Communication	3.63 ± 0.58	3.63 (3.25–4.00)	2.63	3.63	<0.05	4.33 ± 0.48	4.44 (4.00–4.75)	3.38	5.00	<0.05
CCS-Sensitivity	3.86 ± 0.73	3.80 (3.20–4.40)	2.40	5.00	<0.05	4.51 ± 0.46	4.60 (4.20–4.80)	3.40	5.00	<0.05
CCS-Insight	3.39 ± 0.75	3.50 (2.75–4.00)	2.00	4.75	<0.05	4.14 ± 0.55	4.25 (3.75–4.50)	3.00	5.00	<0.05

**Figure 1:**
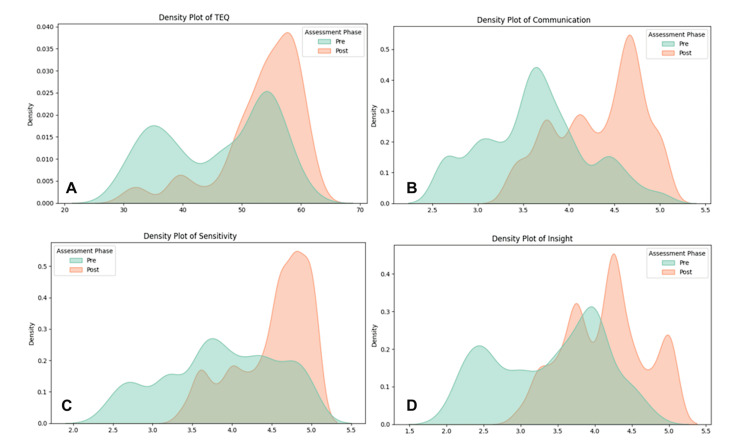
Density plots of TEQ and CCS measurements per assessment phase (pre and post). The figure illustrates the comparative density distributions between the pre-phase (baseline) and post-phase (after intervention) assessments. Panel (A) displays the Toronto Empathy Questionnaire (TEQ) [17] scores. Panels (B), (C), and (D) represent the subscales of the Compassion Competence Scale (CCS) [18]: Communication, Sensitivity, and Insight, respectively. Across all domains, a distinct rightward shift in the curves is observed, indicating a significant enhancement in participants' empathic capacity and compassionate skills. The convergence of the distributions in the post-phase reflects a more uniform attainment of high competency levels across the student cohort. Sources: The Toronto Empathy Questionnaire (TEQ) is adapted from Spreng et al. (2009) [17], and the Compassion Competence Scale (CCS) is adapted from Lee & Seomun (2016) [18]. The validated Greek versions [15,16] were utilized with written permission obtained from the original authors.

Post-intervention assessments revealed a notable shift toward higher empathy ranges, reflecting an overall enhancement in emotional capacity (Figure 1A). A statistically significant increase was observed in the proportion of participants demonstrating higher-than-normal empathy levels. Compassion competence also improved to high levels across all subscales, characterized by marked upward shifts in density distributions for Communication, Sensitivity, and Insight (Figures 1B-D). These findings indicate that the intervention was associated with measurable improvements in both empathic capacity and compassionate professional competence. Formal normality testing confirmed that the data distribution deviated significantly from normality for all scales. Consequently, non-parametric procedures were employed for the subsequent inferential analysis to ensure statistical rigor (Table 1).

Descriptive statistics for the CCS items revealed a consistent upward shift in participants' self-reported competence across the Communication, Sensitivity, and Insight subscales (Table 2).

**Table 2:** Descriptive analysis of individual items of the CCS. Sources: The original Compassion Competence Scale (CCS) is adapted from Lee & Seomun (2016) [18], and the validated Greek version is by Klimentidou et al. (2021) [15]. English item descriptions are provided for reference purposes. Written permission for the use of the validated Greek scale was obtained from the authors.

	Pre-Phase				Post-Phase			
	Mean (±SD)	Md (IQR)	Min	Max	Mean (±SD)	Md (IQR)	Min	Max
I can express my compassion toward patients through communication with them.	3.88 ± 0.66	4.00 (3.00–4.00)	3.00	5.00	4.58 ± 0.57	5.00 (4.00–5.00)	3.00	5.00
I am aware of how to communicate with patients to encourage them.	3.60 ± 0.78	4.00 (3.00–4.00)	1.00	5.00	4.32 ± 0.71	4.00 (4.00–5.00)	3.00	5.00
In conversation, I have a sense of humor to induce a good mood in patients.	3.62 ± 0.75	4.00 (3.00–4.00)	2.00	5.00	4.18 ± 0.72	4.00 (4.00–5.00)	2.00	5.00
Patients express their concerns and difficulties about diseases to me.	3.60 ± 0.81	4.00 (3.00–4.00)	2.00	5.00	4.22 ± 0.68	4.00 (4.00–5.00)	3.00	5.00
I try to support patients to help them overcome their problems.	4.04 ± 0.83	4.00 (4.00–5.00)	2.00	5.00	4.56 ± 0.54	5.00 (4.00–5.00)	3.00	5.00
When communicating with patients, I respond to them with proper nonverbal presentation.	3.52 ± 0.86	3.50 (3.00–4.00)	1.00	5.00	4.20 ± 0.70	4.00 (4.00–5.00)	3.00	5.00
I participate in education to develop interpersonal relationship skills with patients and colleagues.	3.50 ± 1.11	3.50 (3.00–4.00)	1.00	5.00	4.42 ± 0.73	5.00 (4.00–5.00)	3.00	5.00
I can provide the required emotional support to patients appropriately.	3.24 ± 0.85	3.00 (3.00–4.00)	2.00	5.00	4.12 ± 0.80	4.00 (3.00–5.00)	3.00	5.00
I am careful in my speech and behaviors so as to avoid hurting my patient's feelings.	4.12 ± 0.94	4.00 (3.00–5.00)	2.00	5.00	4.68 ± 0.47	5.00 (4.00–5.00)	4.00	5.00
I always pay attention to what patients say.	4.02 ± 0.94	4.00 (3.00–5.00)	2.00	5.00	4.48 ± 0.68	5.00 (4.00–5.00)	3.00	5.00
I promptly respond to patients when they ask for attention.	3.82 ± 0.80	4.00 (3.00–4.00)	2.00	5.00	4.54 ± 0.61	5.00 (4.00–5.00)	3.00	5.00
I am tolerant of others' opinions.	3.84 ± 0.89	4.00 (3.00–4.00)	2.00	5.00	4.48 ± 0.58	5.00 (4.00–5.00)	3.00	5.00
I am well aware of changes in patients' emotional condition.	3.50 ± 0.84	3.00 (3.00–4.00)	2.00	5.00	4.36 ± 0.72	4.50 (4.00–5.00)	3.00	5.00
I am intuitive about patients because of my diverse clinical experience.	2.82 ± 0.87	3.00 (2.00–4.00)	1.00	4.00	3.36 ± 0.92	3.00 (3.00–4.00)	2.00	5.00
I offer customized care to patients by taking their characteristics into consideration.	3.38 ± 0.90	3.50 (3.00–4.00)	2.00	5.00	4.34 ± 0.66	4.00 (4.00–5.00)	3.00	5.00
I look after patients without being influenced by personally challenging situations.	3.76 ± 1.08	4.00 (3.00–5.00)	2.00	5.00	4.44 ± 0.61	4.50 (4.00–5.00)	3.00	5.00
I can empathize well with patients' difficulty	3.58 ± 0.88	4.00 (3.00–4.00)	2.00	5.00	4.40 ± 0.67	4.50 (4.00–5.00)	3.00	5.00

Within the Communication subscale, the most pronounced improvements were observed in students' perceived ability to express compassion and encourage patients through verbal interaction. At baseline, items reflecting non-verbal presentation and the provision of emotional support received the lowest endorsement; however, following the intervention, these scores shifted toward a higher central tendency with significantly reduced dispersion. Notably, participants reported a greater willingness to participate in skills development education and felt more capable of facilitating an environment where patients could openly express their concerns (Table 2).

In the Sensitivity subscale, items generally exhibited high baseline values, which further increased post-intervention. Participants demonstrated a more consistent focus on careful speech and behavior to avoid distressing patients, alongside improved promptness in responding to patient requests. Awareness of changes in patients' emotional conditions, which was initially rated lower compared to other sensitivity items, showed a marked shift toward higher ratings, suggesting enhanced clinical vigilance (Table 2).

The Insight subscale, while presenting the lowest initial ratings and the widest variability, showed substantial developmental gains. Students felt more equipped to offer customized care by considering individual patient characteristics and demonstrated a greater capacity to maintain professional compassion despite personally challenging situations. Although clinical intuition based on experience remained the lowest-rated item, likely due to the students' undergraduate status, the overall distribution moved toward higher values, reflecting an improved empathic understanding of patients' difficulties (Table 2).

Descriptive statistics for the TEQ items demonstrated a generalized improvement in participants' empathic responsiveness following the intervention (Table 3).

**Table 3:** Descriptive analysis of individual items of the TEQ. Sources: The original Toronto Empathy Questionnaire (TEQ) is adapted from Spreng et al. (2009) [17] and the validated Greek version by Kourmousi et al. (2017) [16]. English item descriptions are provided for reference purposes. Written permission for the use of the validated Greek scale was obtained from the authors.

	Pre-Phase				Post-Phase			
	Mean (±SD)	Md (IQR)	Min	Max	Mean (±SD)	Md (IQR)	Min	Max
When someone else is feeling excited, I tend to get excited too.	2.56 ± 0.81	2.00 (2.00–3.00)	1.00	4.00	3.32 ± 0.74	3.00 (3.00–4.00)	2.00	4.00
Other people’s misfortunes do not disturb me a great deal. (R)	2.44 ± 0.73	2.00 (2.00–3.00)	1.00	4.00	2.82 ± 0.98	3.00 (2.00–4.00)	0.00	4.00
It upsets me to see someone being treated disrespectfully.	3.14 ± 0.81	3.00 (3.00–4.00)	1.00	4.00	3.70 ± 0.58	4.00 (3.75–4.00)	2.00	4.00
I remain unaffected when someone close to me is happy. (R)	2.88 ± 0.90	3.00 (2.00–4.00)	0.00	4.00	3.00 ± 1.11	3.00 (3.00–4.00)	0.00	4.00
I enjoy making other people feel better.	3.28 ± 0.86	4.00 (3.00–4.00)	1.00	4.00	3.70 ± 0.54	4.00 (3.00–4.00)	2.00	4.00
I have tender, concerned feelings for people less fortunate than me	2.72 ± 1.01	3.00 (2.00–4.00)	0.00	4.00	3.42 ± 0.70	4.00 (3.00–4.00)	2.00	4.00
When a friend starts to talk about his/her problems, I try to steer the conversation towards something else. (R)	3.02 ± 0.98	3.00 (2.00–4.00)	1.00	4.00	3.24 ± 1.02	3.50 (3.00–4.00)	0.00	4.00
I can tell when others are sad even when they do not say anything	2.74 ± 0.69	3.00 (2.00–3.00)	1.00	4.00	3.34 ± 0.69	3.00 (3.00–4.00)	2.00	4.00
I find that I am “in tune” with other people’s moods.	2.34 ± 0.77	2.00 (2.00–3.00)	1.00	4.00	2.98 ± 0.87	3.00 (2.00–4.00)	1.00	4.00
I do not feel sympathy for people who cause their own serious illnesses. (R)	2.88 ± 0.92	3.00 (2.00–4.00)	1.00	4.00	3.26 ± 0.90	3.00 (3.00–4.00)	0.00	4.00
I become irritated when someone cries. (R)	3.06 ± 0.89	3.00 (2.00–4.00)	1.00	4.00	3.12 ± 0.82	3.00 (3.00–4.00)	0.00	4.00
I am not really interested in how other people feel. (R)	3.18 ± 0.92	3.00 (3.00–4.00)	1.00	4.00	3.66 ± 0.69	4.00 (3.75–4.00)	1.00	4.00
I get a strong urge to help when I see someone who is upset.	2.80 ± 0.88	3.00 (2.00–3.00)	1.00	4.00	3.46 ± 0.76	4.00 (3.00–4.00)	1.00	4.00
When I see someone being treated unfairly, I do not feel very much pity for them. (R)	2.92 ± 1.03	3.00 (2.00–4.00)	1.00	4.00	3.36 ± 0.92	4.00 (3.00–4.00)	0.00	4.00
I find it silly for people to cry out of happiness. (R)	3.02 ± 1.12	4.00 (2.00–4.00)	1.00	4.00	3.26 ± 0.96	4.00 (3.00–4.00)	0.00	4.00
When I see someone being taken advantage of, I feel kind of protective towards him/her.	2.84 ± 0.91	3.00 (2.00–3.00)	0.00	4.00	3.58 ± 0.64	4.00 (3.00–4.00)	2.00	4.00

The most notable gains were observed in items reflecting emotional contagion and attunement (Items 1 and 4), with students reporting a significantly higher tendency to resonate with others' excitement and moods. Emotion comprehension (Item 8), specifically the sensitivity to unexpressed sadness, also showed a marked upward shift, with post-phase responses excluding the lower-scale values observed at baseline. Furthermore, participants exhibited stronger sympathetic physiological arousal (Items 3, 6, 9, and 11), moving from neutral baseline perceptions to a more concentrated emotional reactivity toward the disrespectful or unfair treatment of others (Table 3).

Regarding con-specific altruism and prosocial motivation (Items 5, 13, 14, and 16), there was a clear increase in the "urge to help" when witnessing someone upset, alongside a heightened sense of protectiveness toward individuals being taken advantage of. Tender and concerned feelings for less fortunate individuals also transitioned to higher central values, reflecting enhanced altruistic resonance.

Finally, behaviors indexing sensitivity and situational indifference (Items 2, 7, 10, 12, and 15) showed a positive distribution shift. While responses to items regarding emotional detachment, such as irritation when others cry or dismissive attitudes toward others' problems, remained relatively stable in their central tendency, there was a visible reduction in variability. Post-intervention distributions across these items suggest that the training helped mitigate judgmental attitudes and improved the frequency of appropriate empathic behaviors in clinical scenarios (Table 3).

Inferential analysis

The analysis revealed a statistically significant increase in total empathy scores (TEQ), characterized by a large effect size, suggesting practical significance beyond statistical probability. Similarly, every subscale of the CCS (Communication, Sensitivity, and Insight) demonstrated robust and significant improvements, all yielding large effect size estimates. These findings confirm that the intervention led to a substantial enhancement in both empathic capacity and compassionate professional behaviors across the entire pilot study population (Table 4). Subsequently, the relationship between baseline and post-intervention performance was explored using Spearman's rho correlation. A significant positive correlation was observed for TEQ scores, suggesting that participants' relative standing in empathy remained somewhat consistent despite the overall improvement. In contrast, no significant correlations were found for the Communication, Sensitivity, and Insight subscales. This lack of correlation indicates that the intervention's impact on compassion competence was independent of the students' initial baseline levels, effectively benefiting participants regardless of their starting proficiency (Table 4).

**Table 4:** Non-parametric dependent groups (pre- and post-phase) analysis with the Wilcoxon signed-rank test with effect size estimation, along with non-parametric correlation analysis using Spearman's rho correlation coefficient of TEQ and CCS measurements. Statistical analysis of the pre- and post-intervention scores. Sources: The Toronto Empathy Questionnaire (TEQ) is adapted from Spreng et al. (2009) [17] and the Compassion Competence Scale (CCS) from Lee & Seomun (2016) [18]. The validated Greek versions [15,16] were utilized with written permission obtained from the original authors. The original English items for both instruments are provided in the Appendix for reference.

Post-Phase Measurement–Pre-Phase Measurement	Wilcoxon Signed-Rank Test		Non-parametric Correlation Analysis		Effect Size
	Z	p-value	rho	p-value	r
TEQ	-4.334	<0.05	0.343	<0.05	0.613
CCS-Communication	-5.065	<0.05	0.040	>0.05	0.716
CCS-Sensitivity	-4.653	<0.05	0.228	>0.05	0.658
CCS-Insight	-4.667	<0.05	0.059	>0.05	0.660

Intra-faculty progression

A granular analysis of the intra-departmental data highlights that although all groups demonstrated significant progress, they originated from markedly different baselines. Nursing students commenced the pilot study with the highest baseline empathy scores; nevertheless, they achieved further statistically significant gains post-intervention, with a medium effect size. Their scores in the compassion competence subscales also showed steady and significant improvement, particularly in the Communication domain (Table 5).

**Table 5:** Descriptive statistics and non-parametric dependent groups (pre- and post-phase) analysis with the Wilcoxon signed-rank test with effect size estimation of TEQ and CCS measurements per university faculty group. Analysis of the intra-departmental data. Sources: The Toronto Empathy Questionnaire (TEQ) is adapted from Spreng et al. (2009) [17] and the Compassion Competence Scale (CCS) from Lee & Seomun (2016) [18]. The validated Greek versions [15,16] were utilized with written permission obtained from the original authors. The original English items for both instruments are provided in the Appendix for reference.

University Faculty	Research Measurements	Assessment Phase: Pre-Phase		Assessment Phase: Post-Phase		Wilcoxon Signed-Rank Test		Effect Size
		Mean ± SD	Md (IQR)	Mean ± SD	Md (IQR)	Z	p-value	r
Department of Nursing (n=25)	TEQ	51.56 ± 6.96	53.00 (50.00–56.00)	53.72 ± 5.52	54.00 (50.00–58.00)	-2.078	<0.05	0.416
	CSS-Communication	3.90 ± 0.51	3.88 (3.63–4.13)	4.17 ± 0.37	4.13 (3.88–4.50)	-2.605	<0.05	0.521
	CSS-Sensitivity	4.22 ± 0.52	4.20 (3.80–4.60)	4.42 ± 0.47	4.60 (4.00–4.80)	-1.887	<0.05	0.377
	CSS-Insight	3.83 ± 0.50	4.00 (3.50–4.00)	4.02 ± 0.48	4.00 (3.75–4.25)	-1.781	<0.05	0.356
Department of Physiotherapy (n=15)	TEQ	41.20 ± 9.76	36.00 (34.00–53.00)	49.67 ± 9.72	54.00 (40.00–57.00)	-2.103	<0.05	0.543
	CSS-Communication	3.46 ± 0.60	3.50 (3.00–3.88)	4.31 ± 0.62	4.63 (3.63–4.88)	-3.050	<0.05	0.788
	CSS-Sensitivity	3.69 ± 0.79	3.60 (3.20–4.40)	4.49 ± 0.54	4.60 (4.40–5.00)	-3.020	<0.05	0.780
	CSS-Insight	3.07 ± 0.78	3.00 (2.25–3.75)	4.15 ± 0.71	4.25 (3.50–5.00)	-3.019	<0.05	0.779
Department of Medicine (n=10)	TEQ	38.40 ± 4.62	39.00 (35.00–40.00)	57.30 ± 2.26	58.00 (57.00–59.00)	-2.840	<0.05	0.898
	CSS-Communication	3.20 ± 0.37	3.25 (2.88–3.63)	4.72 ± 0.16	4.69 (4.63–4.75)	-2.807	<0.05	0.888
	CSS-Sensitivity	3.22 ± 0.58	3.20 (2.80–3.60)	4.76 ± 0.16	4.80 (4.60–4.80)	-2.807	<0.05	0.888
	CSS-Insight	2.75 ± 0.51	2.50 (2.50–3.00)	4.40 ± 0.34	4.25 (4.25–4.50)	-2.812	<0.05	0.890

In contrast, students from the Departments of Physiotherapy and Medicine displayed a more pronounced upward trajectory. Physiotherapy students showed a dramatic increase in empathy scores alongside notable improvements across all compassion domains, particularly in Sensitivity and Insight, both yielding high effect sizes.

The most substantial shift was observed in the Medical department. Medical students entered the pilot study with the lowest initial median scores in both empathy and compassion competence but reached the highest post-intervention levels among all groups, representing very large effect sizes across all measured parameters. Their compassion competence scores saw a remarkable standardization at high levels, specifically in Communication and Sensitivity (Table 5).

This post-intervention convergence suggests that the asynchronous online platform acted as an equalizing educational tool. It effectively standardized compassion competence levels across diverse healthcare disciplines, regardless of the participants' academic background or their initial humanistic orientation. By bridging these baseline disparities, the intervention fostered a unified level of professional readiness across all faculties (Table 5).

In summary, the intra-faculty analysis demonstrated a universal improvement across all student groups, regardless of their academic background. Notably, the most pronounced intervention effect was observed in the Departments of Medicine and Physiotherapy, where students originated from significantly lower baseline empathy and compassion scores compared to their Nursing peers. By the conclusion of the intervention, these groups achieved score levels comparable to those of the Nursing faculty, effectively bridging the initial competency gap. This observed convergence, supported by the large effect sizes observed in the non-parametric analysis, suggests that the asynchronous digital format may serve as a standardized preparatory foundation for humanistic competence across diverse health science disciplines.

## Discussion

The present pilot study aimed to evaluate the impact of an asynchronous digital intervention on enhancing empathy and compassion competence among healthcare students. Our findings demonstrate a significant and robust improvement across all measured domains following the intervention. Specifically, participants showed a substantial increase in emotional empathy, as well as improved skills in compassionate communication, sensitivity to others' suffering, and clinical insight. A more granular analysis of individual items confirmed that these improvements were particularly evident in behavioral responsiveness and clinical etiquette. Furthermore, the intervention appeared to bridge the initial gap between different healthcare disciplines, fostering a more uniform level of humanistic competence. These results suggest that structured, narrative-driven digital education can effectively address current curricular deficiencies in healthcare humanism.

The significant enhancement observed highlights the potential of asynchronous digital education to mitigate the humanistic deficit in modern healthcare. As Lown (2016) suggests, we are witnessing a global compassion crisis in health systems, making scalable digital interventions a necessity rather than an elective addition to curricula [19]. The robust gains in the "Sensitivity" and "Communication" subscales indicate that digital tools can facilitate what Terry and Cain (2016) conceptualize as "Digital Empathy" [20]. By utilizing narrative-driven animations, the platform bypasses technological barriers, creating a space where students can practice emotional resonance. This aligns with Riess (2017), who posits that empathy is a neurobiological capacity that can be "re-wired" through repetitive, high-fidelity stimuli, such as those provided by our digital storytelling modules [2].

The empathy erosion documented by Neumann et al. (2011) is often a defense mechanism against empathetic distress [6]. As Decety (2011) notes, without proper training, healthcare students may engage in emotional disengagement as a regulatory strategy to survive the clinical environment [21]. Our intervention addresses this by teaching compassion as a proactive skill. Unlike pure empathy, which can lead to burnout, compassion involves a prosocial motivation to act [22]. While not directly measured in this study, the literature suggests that the cultivation of compassion competence could potentially serve as a protective factor against burnout and moral distress. By fostering "Insight" and "Self-Compassion" [23], the intervention builds moral resilience, allowing students to remain emotionally open without succumbing to compassion fatigue [24,25].

The "convergence effect" observed, where students from disciplines traditionally dominated by the biomedical model showed the highest rates of improvement, suggests that while Nursing curricula traditionally emphasize the "ethic of care" [26], medical and physiotherapy students possess a latent humanistic capacity that only requires a structured stimulus to manifest. This supports Hojat's (2018) view that empathy is a cognitive attribute that can be taught [5]. By standardizing these competencies, our digital tool acts as a standardized preparatory foundation that fosters a unified inter-professional language of compassion, the cornerstone of safe, patient-centered care [8]. Through narrative medicine, students developed the ability to "honor the stories of illness" [11]. This practice activates perspective-taking, which Ekman (2003) identifies as essential for clinical encounters [27]. The asynchronous format further supported transformative learning [13] by allowing for reflective pauses, which are critical for shifting professional identity from a technician to a compassionate professional.

The exceptionally large effect size observed in medical students suggests that digital narrative-based tools can act as a powerful catalyst for students with limited prior exposure to humanistic training. While the findings provide a strong signal for the intervention’s potential, they should be viewed as preliminary and subject to verification in larger-scale trials. This pilot study was an exploratory step to test the feasibility of digital compassion training. While the current study focused on student competence, the ultimate goal of such interventions is the improvement of objective patient outcomes. However, within the scope of our data, these links remain hypothetical. Future longitudinal research should prioritize the investigation of whether these gains in compassion competence translate into measurable benefits for patient care, such as increased patient satisfaction, improved treatment adherence, and enhanced clinical safety.

Strengths and limitations

A significant strength of this pilot study is the high psychometric robustness of the instruments used. Internal consistency reliability was verified for all measures at both assessment phases using Cronbach’s alpha. In the pre-phase, the TEQ demonstrated excellent internal consistency (α=0.91), while the CCS subscales showed good reliability for Communication (α=0.84), Sensitivity (α=0.88), and Insight (α=0.81). In the post-phase, reliability remained within acceptable thresholds (TEQ: α=0.84; CCS subscales: α=0.75-0.85), supporting the validity of the post-intervention assessments and the overall stability of the findings.

Despite these strengths, this study is subject to several limitations inherent to its pilot nature. First, the study utilized a convenience sampling technique, with the sample size determined by voluntary enrollment during a specific academic period without an a priori power analysis. While this ensured relevance to the research objectives, the geographical and institutional homogeneity may limit the generalizability (external validity) of the findings to educational contexts outside of Greece.

Second, the exclusive reliance on self-reported psychometric instruments introduces the potential for social desirability bias. Students may have subconsciously reported higher levels of empathy to align with perceived professional expectations. Although the anonymity of the digital platform was intended to mitigate this risk, the findings represent the students' perceived competence rather than objective behavioral change.

Third, the absence of a control group and the lack of longitudinal follow-up preclude the ability to definitively isolate the intervention's effect from natural maturation or to assess the sustainability of improvements once students transition into "systemically uncompassionate" clinical environments [28]. Additionally, the moderate-to-high baseline scores observed in the Nursing subgroup suggest a potential ceiling effect, which may have constrained the statistical margin for further improvement.

Finally, the small sample sizes in the Schools of Medicine and Physiotherapy preclude definitive conclusions regarding interdisciplinary differences. Moreover, while the asynchronous nature of the platform ensured flexibility and scalability, it lacked the real-time interpersonal interaction and role-playing characteristic of traditional workshops.

Implications for future research

Future research should prioritize longitudinal designs to evaluate the decay rate of these competencies as students transition from the digital classroom to the clinical bedside. Understanding whether gains in compassion competence translate into measurable changes in bedside behavior and, eventually, into objective patient outcomes such as satisfaction, diagnostic accuracy, and treatment adherence is a critical next step to validate the clinical utility of the intervention.

Moreover, given the global shift toward frameworks such as the AETCOM modules, future studies should investigate how digital narrative tools can be systematically embedded across various curriculum phases. Such research could clarify whether digital interventions effectively support the ongoing development of professional identity from pre-clinical years to bedside practice. To minimize confounding variables, future trials could employ stricter inclusion criteria, such as selecting students at the same academic stage to account for varying levels of prior clinical exposure.

Methodologically, future research should move beyond self-reported data by incorporating objective performance metrics or workplace-based assessments. Future iterations of this program could also explore a "blended" learning model, combining the scalability of asynchronous digital modules with synchronous peer-to-peer dialogue and role-playing to foster deeper interpersonal resonance.

Lastly, the role of emerging technologies, such as virtual reality (VR) and generative artificial intelligence (AI), should be explored to enhance the empathetic immersion of digital scenarios. These tools could provide sophisticated, high-fidelity simulations that challenge students' narrative competence and emotional regulation in increasingly complex and high-pressure clinical environments.

## Conclusions

In conclusion, this pilot study highlights a significant educational gap, as the vast majority of healthcare students lacked prior formal training in compassionate care. Our findings provide preliminary evidence that an asynchronous digital platform can serve as a potentially beneficial and scalable proof-of-concept for delivering humanistic education, as it was associated with encouraging gains in empathy and compassion competence across all participants. The intervention's success in fostering the development of these skills among students from different disciplines suggests that digital pedagogy is a viable tool for bridging competency gaps and fostering a unified inter-professional language of care. However, given the pilot nature of the design and the absence of a control group, these results should be interpreted as a promising signal rather than definitive proof of efficacy.

## Appendices

Toronto Empathy Questionnaire (TEQ)

Instructions: Below is a list of statements. Please rate how often you feel this way by using the following scale:

0 = Never; 1 = Rarely; 2 = Sometimes; 3 = Often; 4 = Always

**Table 6:** The Toronto Empathy Questionnaire (TEQ). The Toronto Empathy Questionnaire (TEQ) [17]. Scoring: Item responses are scored according to the following scale for positively worded items 1, 3, 5, 6, 8, 9, 13, 16. Never = 0; Rarely = 1; Sometimes = 2; Often = 3; Always = 4. The following negatively worded items are reverse-scored: 2, 4, 7, 10, 11, 12, 14, 15. Never = 4; Rarely = 3; Sometimes = 2; Often = 1; Always = 0. Scores are summed to derive the total for the TEQ.

No.	Item Description	Never	Rarely	Sometimes	Often	Always
1	When someone else is feeling excited, I tend to get excited too.	0	1	2	3	4
2	Other people’s misfortunes do not disturb me a great deal. (R)	0	1	2	3	4
3	It upsets me to see someone being treated disrespectfully.	0	1	2	3	4
4	I remain unaffected when someone close to me is happy. (R)	0	1	2	3	4
5	I enjoy making other people feel better.	0	1	2	3	4
6	I have tender, concerned feelings for people less fortunate than me.	0	1	2	3	4
7	When a friend starts to talk about his/her problems, I try to steer the conversation towards something else. (R)	0	1	2	3	4
8	I can tell when others are sad even when they do not say anything.	0	1	2	3	4
9	I find that I am “in tune” with other people’s moods.	0	1	2	3	4
10	I do not feel sympathy for people who cause their own serious illnesses. (R)	0	1	2	3	4
11	I become irritated when someone cries. (R)	0	1	2	3	4
12	I am not really interested in how other people feel. (R)	0	1	2	3	4
13	I get a strong urge to help when I see someone who is upset.	0	1	2	3	4
14	When I see someone being treated unfairly, I do not feel very much pity for them. (R)	0	1	2	3	4
15	I find it silly for people to cry out of happiness. (R)	0	1	2	3	4
16	When I see someone being taken advantage of, I feel kind of protective towards him/her.	0	1	2	3	4

Compassion Competence Scale (CCS)

Instructions: Read the following 17 items and select the response that best applies to you. Respond to all items based on the following scale:

**Table 7:** Compassion Competence Scale (CCS). The Compassion Competence Scale (CCS) [18]. Scoring Information: The total score is calculated as the average value of all 17 items. The scale measures three dimensions: Communication (items 1-8), Sensitivity (items 9-13), and Insight (items 14-17).

No.	Item Description	1	2	3	4	5
1	I can express my compassion toward patients through communication with them.	1	2	3	4	5
2	I am aware of how to communicate with patients to encourage them.	1	2	3	4	5
3	In conversation, I have a sense of humor to induce a good mood in patients.	1	2	3	4	5
4	Patients express their concerns and difficulties about diseases to me.	1	2	3	4	5
5	I try to support patients to help them overcome their problems.	1	2	3	4	5
6	When communicating with patients, I respond to them with proper nonverbal presentation.	1	2	3	4	5
7	I participate in education to develop interpersonal relationship skills with patients and colleagues.	1	2	3	4	5
8	I can provide the required emotional support to patients appropriately.	1	2	3	4	5
9	I am careful in my speech and behaviors so as to avoid hurting my patient's feelings.	1	2	3	4	5
10	I always pay attention to what patients say.	1	2	3	4	5
11	I promptly respond to patients when they ask for attention.	1	2	3	4	5
12	I am tolerant of others' opinions.	1	2	3	4	5
13	I am well aware of changes in patients' emotional condition.	1	2	3	4	5
14	I am intuitive about patients because of my diverse clinical experience.	1	2	3	4	5
15	I offer customized care to patients by taking their characteristics into consideration.	1	2	3	4	5
16	I look after patients without being influenced by personally challenging situations.	1	2	3	4	5
17	I can empathize well with patients' difficulty.	1	2	3	4	5

All items reproduced for academic reference purposes from [17,18]. The validated Greek versions [15,16] were utilized with written permission obtained from the original authors.
